# Leveraging neuroscience education to address stigma related to opioid use disorder in the community: a pilot study

**DOI:** 10.3389/fpsyt.2024.1360356

**Published:** 2024-03-18

**Authors:** Evan J. Kyzar, Melissa R. Arbuckle, Adam Abba-Aji, Krishna Balachandra, Joseph Cooper, Adriane Dela Cruz, Ellen Edens, Brady Heward, Michael Jibson, Ayana Jordan, Daniel Moreno-De-Luca, Hannah Pazderka, Mohit Singh, Jeremy J. Weleff, Bernice Yau, Justin Young, David A. Ross

**Affiliations:** ^1^ Department of Psychiatry, Columbia University Irving Medical Center, New York State Psychiatric Institute, New York, NY, United States; ^2^ Department of Psychiatry, University of Alberta Faculty of Medicine and Dentistry, Edmonton, AB, Canada; ^3^ Department of Psychiatry, University of Illinois, Chicago, IL, United States; ^4^ Department of Psychiatry, University of Texas, Dallas, TX, United States; ^5^ Department of Psychiatry, Yale University School of Medicine, New Haven, CT, United States; ^6^ Department of Psychiatry, University of Vermont Larner College of Medicine, Burlington, VT, United States; ^7^ Department of Psychiatry, University of Michigan, Ann Arbor, Michigan MI, United States; ^8^ Department of Psychiatry, New York University School of Medicine, New York, NY, United States; ^9^ CASA Mental Health, Edmonton, AB, Canada; ^10^ Department of Psychiatry and Psychology, Center for Behavioral Health, Neurological Institute, Cleveland Clinic, Cleveland, OH, United States

**Keywords:** opioids, addiction, stigma, neuroscience, education, community health

## Abstract

Opioid use disorder (OUD) and overdose deaths are a public health crisis. One contributing factor is stigma towards people who use opioids. We developed and conducted a public-facing, half-day educational event designed to challenge misperceptions about OUD from a contemporary neuroscience perspective. Participants engaged with three different resources on the neurobiology of addiction, and, at the end of the event, they rated its effectiveness. We also collected and compared pre- and post-event composite OUD stigma scales. Participants rated our approach and the overall event as highly effective. Additionally, OUD stigma scores were lower immediately following the event, and this decrease was primarily driven by decreased internalized stigma. Here, we demonstrate an effective proof-of-concept that an accessible, public-facing, neuroscience education event may reduce OUD stigma in the community.

## Introduction

North America is in the midst of an overdose crisis, with fatal opioid overdoses reaching over 75,000 per year in 2020 (1) and rising. This crisis has had devastating effects across socioeconomic groups, with disproportionate impacts on historically marginalized communities due to inequitable systems of care (2). There is an urgent need for interventions that address opioid use disorder (OUD) not only with patients and their family members, but also in the broader community.

Multiple factors have contributed to the current crisis, including the proliferation of synthetic opioids, limited access to evidence-based interventions for OUD, and social determinants of health. Another key contributor is stigma, defined as the association of negative characteristics or stereotypes against individuals labeled as belonging to a particular group (3). Stigma towards people who use drugs, in large part the legacy of moral conceptualizations that invite blame and prejudice towards the user, can become particularly insidious when these stigmatized beliefs become internalized as part of a self-narrative (4). For individuals with OUD, internalized negative beliefs may limit their engagement with healthcare systems (5, 6), leading to poorer treatment outcomes. Similarly, externalized stigma among clinicians, especially those without specialty training in substance use disorders (SUDs), may prevent them from implementing evidence-based approaches if they subconsciously blame patients or otherwise misunderstand SUDs, treatment, and recovery (7, 8). In the general population, increased stigma towards people who use opioids is associated with lower levels of support for addiction services (9). Despite this, relatively few studies have directly tested the impact of internalized or perceived stigma on treatment outcomes in OUD patients (10).

One approach to addressing stigma is to combat ignorance and fear with understanding and hope – as has happened in other branches of medicine, such as cancer (11). For many years, our understanding of the biology of SUDs was limited by available scientific approaches. Modern neuroscience has enabled a robust understanding of addiction: how both genetic and environmental factors can translate into vulnerability or resilience through shared neurobiological pathways. These insights have, in part, led to an evolution in explanatory theories of addiction – from a disease of moral failure to one influenced by neuroadaptation and biology (12, 13). As our understanding of addiction shifts, an opportunity exists for educational interventions that harness these new findings in a way that can decrease stigma and enhance engagement with treatment. While there have been a small number of studies on various methods to decrease stigma related to addiction (14), to our knowledge there have been no studies evaluating the effect of neuroscience education on OUD stigma.

To this end, we designed a public-facing educational intervention that was rooted in principles of adult learning. We piloted the approach in a community sample that included individuals with lived experience, family members, and health care providers. While our event was open to all members of the general public, we reasoned that these groups constitute critical stakeholders in any intervention aimed at addressing OUD stigma. Our primary objective was to determine the feasibility and effectiveness of our event. We compared pre- and post-event stigma scores retrospectively to assess the impact on participant attitudes.

## Methods

### Event recruitment and setting

The 3-hour educational event was held on March 20^th^, 2023 at the University of Alberta in Edmonton, Canada. Participants were recruited via flyers in the local area (posted on the University of Alberta campus and at clinical sites in Edmonton), outreach to community organizations (e.g., Moms Stop the Harm, Families supporting Adults with Mental Illness [FAMI]), and posts on social media. We advertised that we were developing an educational program on OUD and wanted feedback from people with lived and living experience, family members, and the broader public. Following standards for community engagement, participants were compensated for their time (with a 100 CAD gift card) and breakfast and lunch were provided. Participants were informed that their feedback would be used for quality improvement.

### Educational event design and description of intervention

The program was designed around principles of adult learning theory: using experiential learning approaches, facilitating differentiation (consistent with a constructivist model), leveraging social connection among participants, and incorporating formative assessment tools (15, 16). While we focused on neuroscience resources, our event offered a holistic message of hope and recovery. This was accomplished both via messaging from the facilitators during the event and in the neuroscience resources themselves, which specifically mentioned that existing treatments work and that recovery is always possible. The neuroscience resources were additionally designed according to best practices of effective scientific communication (e.g., using narrative approaches to make content broadly accessible, connecting content to real-world scenarios of lived experience, and attending to data visualization). We crafted three vignettes centered around OUD (see **Supplementary Methods** for full vignettes) meant to create personal moments of charged, affective salience for the reader.

**Figure 1:**
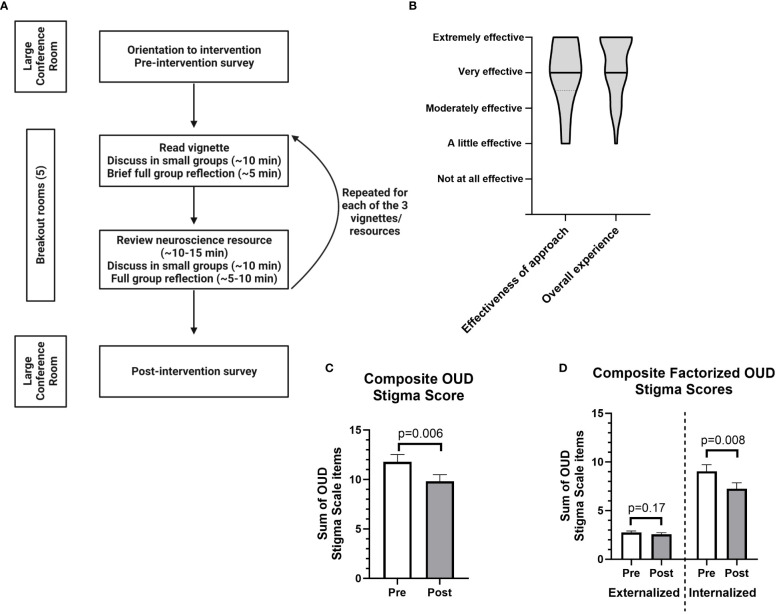
Design of a neuroscience-focused educational event and its perceived effectiveness and impact on stigma scales related to opioid use disorder (OUD). **(A)** Flowchart outlining the organization of the program. **(B)** Data on the perceived effectiveness of the approach and the overall experience of the event, rated on a Likert scale which was numerically converted for graphical representation as follows: Extremely effective = 5; Very effective = 4; Moderately effective = 3; A little effective = 2; Not at all effective = 1. Graphs show smoothed violin plots with black bars indicating median response. **(C)** Pre- and post-event ratings for composite OUD stigma score: Z=-2.74, W=-240, *p*​=​0.006 (primary hypothesis tested at α=0.05). Graph shows mean ± SEM. **(D)** Pre- and post-event ratings for two summed OUD stigma scale factors (termed externalized and internalized) identified using exploratory factor analysis (see **Suppleemntary Figure S1**); Externalized factor (2 questions): Z=-1.44, W=-44, *p*​=​0.017; Internalized factor (3 questions): Z=-2.65, W=-185, *p*​=​0.008 (significant when Bonferroni corrected at α=0.025). Graph shows mean ± SEM. All stigma scales were rated on a Likert scale which was numerically converted for statistical analyses as follows: Strongly agree = 5; Agree = 4; Neither agree nor disagree = 3; Disagree = 2; Strongly disagree = 1. All data were analyzed using Wilcoxon signed-ranks tests with n=37, as the Likert data was not normally distributed. See **Supplementary Table 1** for raw means of pre- and post-event data. Note that the post-event survey was completed approximately 3-3.5 hours after the pre-event survey, immediately following the conclusion of the educational event.

Participants were assigned to five breakout rooms of ~10 participants with 2-3 facilitators per room. For each of the three vignettes (**Figure 1A**) participants worked in groups of 2-3 to discuss their initial responses to the scenario, review a neuroscience-focused educational resource, and then reflect on how the resource might change the way they thought about the scenario. The resources were crafted to highlight core questions – and misconceptions – relating to OUD: 1) a video on genetic and environmental contributions to risk (17); 2) a short article on long-term affective changes that contribute to return to opioid use after abstinence (18); and 3) a video on the contribution of negative affective states to opioid use (19). Following each small group activity there was a brief full group discussion with summary of key themes.

### Data collection

Participants filled out a pre-survey that included: self-identification of whether they or their family member(s) had lived experience with addiction and whether they worked in health care (in order to maintain anonymity and encourage participation, we minimized the number of questions asked and did not collect any individually identifying data); five Likert scale questions derived from the Opening Minds Stigma Scale (20) modified for a community sample; and open-ended responses to the three case vignette prompts (see **Supplementary** Methods for details).

Immediately after the breakout room activities, participants were asked to complete a survey that included the same questions as the pre-survey plus additional questions about the effectiveness of our approach and their overall experience of the event.

### Statistical analyses

Our data analyses were approved by the Research Ethics Board (REB) at the University of Alberta. We did not perform an *a priori* power analysis, though a *post hoc* analysis revealed that a sample size of 34 would be sufficient to detect a moderate effect (*d*=0.05). Likert scale responses were converted to numerical data. We examined the correlation between participants’ rating of the effectiveness of approach and overall experience using a Pearson test. We tested internal consistency of the five OUD stigma questions with Cronbach’s alpha (α = 0.75) and performed exploratory factor analysis given that this scale has been primarily used in healthcare providers and not the general population. These data were summed to create a composite OUD stigma score. Our main hypothesis related to retrospective analysis of OUD stigma scores was that the program would decrease composite stigma scores (α=0.05). We performed secondary analyses on the identified factors from the factor analysis (created by summing questions from each of the factors) and each stigma question with Bonferroni correction for multiple comparisons (α=0.025 for factors; α=0.01 for each question). We used nonparametric Wilcoxon signed-rank tests for these analyses. We examined the change in composite OUD stigma score depending on group self-identification (by Mann-Whitney *U*-test) and depending on ratings of the effectiveness of approach and overall experience of the event (by one-way ANOVA). We additionally examined whether ratings of effectiveness of approach or overall experience changed based on group self-identification (by Mann-Whitney *U*-test). Data were analyzed in R v4.0.4 and GraphPad Prism 9.

## Results

### Participant ratings of effectiveness and overall experience

Overall, 47 unique participants from the community attended the event (**Figure 1A**), and 37 participants completed both the pre- and post-event formative assessment (78.72%). Amongst participants who completed both surveys, 16/37 (43.24%) had lived experience with addiction, 24/37 (64.86%) had a family member with lived experience of addiction, and 17/37 (45.95%) were employed as a health care worker. These categories were not mutually exclusive, with 19/37 (51.35%) participants answering affirmatively to ≥2 of these categories and 3/37 (8.11%) answering in the negative to all. Participants found both the approach and the overall experience to be effective (**Figure 1B**), and ratings of the effectiveness of our approach and the overall experience were highly correlated (R^2^ = 0.6492, *p*<0.0001).

### Effect on OUD stigma scores

We performed a retrospective analysis of stigma scores collected pre- and post-event. Factor analyses of our adapted stigma scale revealed two factors related to externalized stigma (i.e., towards others) and internalized stigma (i.e., self-stigma), respectively (**Supplementary Figure S1**). For example, the prompt “I struggle to feel compassion for a person with opioid use disorder” was in the externalized factor, while “I would see myself as weak if I had opioid use disorder and could not fix it myself” was in the internalized factor. This factor structure was consistent with the identified factors of “Attitude” (similar to externalization of stigma) and “Disclosure/Help Seeking” (similar to internalization of stigma) from the Opening Minds Stigma Scale (20).

We found that summed post-event composite OUD stigma scores were significantly lower compared to pre-event scores (Z=-2.74, W=-240, *p*​=​0.006) (**Figure 1C**), despite the observation that pre-event scores were relatively low in our sample. We next performed secondary analyses to determine if particular factors or questions may be driving the decrease in post-event composite scores. Summed internalized stigma questions identified in the factor analysis were decreased post-event (Z=-2.65, W=-185, *p*=0.008) with no effect on questions related to externalized stigma (**Figure 1D**). Raw mean stigma scores decreased for all five questions (**Supplementary Figure S2**, **Supplementary Table 1**). Interestingly, the two largest decreases in individual questions came on questions related to internalized or self-stigma (**Supplementary Figures S2D, E**), though neither met stringent criteria for statistical significance after correction for multiple comparisons. Raw Likert scale responses for pre- and post-event surveys are shown in **Supplementary Figure S3**.

### Effect of group self-identification on change in composite OUD stigma scores

We tested whether self-identifying as belonging to particular groups had an effect on the change in composite OUD stigma scores between pre- and post-event surveys, finding that there were no differences between participants with and without lived experience (**Supplementary Figure S4A**), participants with and without family member(s) with lived experience (**Supplementary Figure S4B**), and health care workers versus non-health care workers (**Figure S4C**). Identification with these groups had no effect on ratings of the overall experience or effectiveness of the event (**Supplementary Figures S4D–F**). Additionally, the change in composite OUD stigma scores between pre- and post-event surveys did not depend on ratings of the overall experience (**Supplementary Figure S4G**) or the effectiveness of our approach (**Supplementary Figure S4H**).

## Discussion

Here we describe a proof-of-concept pilot of a public-facing, community-level program focused on addressing OUD stigma using neuroscience education. We designed our intervention with an explicit focus on adult learning theory (15, 16), utilizing accessible resources to create interactive learning opportunities. Given the pilot nature of our event, we were principally interested in individuals’ experience of the event. Participants viewed both our approach and the overall event as highly effective (**Figure 1B**).

We performed a retrospective analysis of survey data to determine whether our event had an effect of OUD stigma. Many of the participants had personal experience with addiction, and stigma scores (particularly for externalized stigma) were relatively low in our sample. Despite this potential floor effect, mean composite OUD stigma scores decreased post-event (**Figure 1C**), an effect which appeared independent of individuals’ prior experience with addiction and/or working in health care. Interestingly, the decrease in OUD stigma scores appears to have been driven by decreases in internalized or self-stigma (**Figure 1D**).

Our pilot event and reported results add to the emerging literature on addressing stigma through educational approaches. While some authors have suggested that neuroscience education may paradoxically increase stigma (21, 22), our results suggest that, at least in our sample, this was not the case. We hypothesize that this may have been due to our overall approach, where neurobiological attributions were incorporated alongside personal vignettes and messages emphasizing the possibility of recovery. Previous research has shown that presenting addiction through the lens of different models or descriptions differentially alters dimensions of stigma towards people who use drugs (23, 24). Specifically, presenting addiction as a ‘chronic relapsing brain disorder’ led to lower ratings of stigmatizing blame, but higher need for continuing care and lower prognostic optimism (23). Therefore, future research should focus on a broader range of stigma dimensions that may not have been fully captured in our brief survey.

There are a number of possible reasons why we observed a decrease in OUD stigma. First, while we highlighted accessible neuroscience resources, our event also conveyed a holistic message of hope that emphasized that effective treatments are available and that recovery is possible. Similar recovery-oriented interventions have been shown to reduce internalized stigma in mental illnesses (25). Additionally, our resources highlighted the combined influences of genetic factors and environmental stressors, as well as the importance of addressing modifiable risk factors in recovery. We speculate that the success of our approach hinged on this balance between calling attention to critical neuroscientific concepts while also conveying hope in a recovery-centered way.

We emphasize that our results represent a proof-of-concept pilot, and as such there are important caveats and limitations. Our sample size was limited, and the participatory nature of the event raises the possibility of sampling biases. This likely contributed to the lower pre-event stigma scores, though we saw a decrease in post-event scores regardless. We minimized questions on personal information to decrease barriers to participation, and it is possible that our intervention had differential effects on specific groups. Additionally, the exact psychological mechanisms that underlie the observed decrease in OUD stigma are unclear. Others factors that may contribute include the impact of increased social support and connection with other participants with similar backgrounds (26, 27), the above-mentioned hopeful and recovery-oriented message, and the use of personal stories from those with lived experience, which have been separately shown to decrease stigma (28).

As this was a community-facing pilot study, future work should attempt to extend these findings to a broader and more heterogenous sample, including individuals with higher levels of baseline stigma. Additionally, though individuals with lived experience did attend our event, this was explicitly not a clinical population. Neuroscience education for other chronic illness such as chronic pain improves functional outcomes (29), and future studies should directly address whether a similar educational strategy may alter treatment outcomes in clinical OUD populations.

## Conclusions

There are numerous strategies that could help combat the ongoing overdose crisis, including improved access to evidence-based treatments, robust harm reduction approaches, and initiatives to address underlying social determinants of health. Novel educational interventions, such as the one outlined in this report, could offer a powerful additional tool by offering a message of hope and recovery, decreasing stigma, enhancing engagement with evidence-based treatments, and facilitating more thoughtful public policies.

## Data availability statement

The raw data supporting the conclusions of this article will be made available by the authors, without undue reservation.

## Ethics statement

The studies involving humans were approved by Research Ethics Board (REB) at the University of Alberta. The studies were conducted in accordance with the local legislation and institutional requirements. The participants were informed that their de-identified data would be used to improve the event and would be disseminated.

## Author contributions

EK: Conceptualization, Data curation, Formal analysis, Investigation, Methodology, Writing – original draft, Writing – review & editing. MA: Conceptualization, Investigation, Methodology, Resources, Supervision, Validation, Writing – original draft, Writing – review & editing. AA: Conceptualization, Investigation, Writing – review & editing. KB: Conceptualization, Investigation, Writing – review & editing. JC: Conceptualization, Investigation, Methodology, Writing – review & editing. AC: Conceptualization, Investigation, Writing – review & editing. EE: Conceptualization, Investigation, Writing – review & editing. BH: Conceptualization, Investigation, Writing – review & editing. MJ: Conceptualization, Investigation, Writing – review & editing. AJ: Conceptualization, Investigation, Writing – review & editing. DM: Conceptualization, Investigation, Writing – review & editing. HP: Data curation, Investigation, Writing – review & editing. MS: Conceptualization, Investigation, Writing – review & editing. JW: Conceptualization, Investigation, Writing – review & editing. BY: Conceptualization, Investigation, Writing – review & editing. JY: Conceptualization, Investigation, Writing – review & editing. DR: Conceptualization, Funding acquisition, Investigation, Methodology, Resources, Supervision, Writing – original draft, Writing – review & editing.
